# Optimization of magnetic properties and GMI effect of Thin Co-rich Microwires for GMI Microsensors

**DOI:** 10.3390/s20061558

**Published:** 2020-03-11

**Authors:** Lorena Gonzalez-Legarreta, Paula Corte-Leon, Valentina Zhukova, Mihail Ipatov, Juan Maria Blanco, Julian Gonzalez, Arcady Zhukov

**Affiliations:** 1 Dpto. Física de Materiales, Facultad de Química, UPV/EHU, Paseo Manuel de Lardizabal, 3, 20018 San Sebastian, Spain; lorena.glegarreta@gmail.com (L.G.-L.); paula.corte@ehu.eus (P.C.-L.); valentina.zhukova@ehu.es (V.Z.); mihail.ipatov@ehu.es (M.I.); 2 Dpto. QUIPRE, Inorganic Chemistry-University of Cantabria, Nanomedice-IDIVAL, Avda. de Los Castros 46, 39005 Santander, Spain; 3 Dpto. de Fisica Aplicada, EIG, Basque Country University (UPV/EHU), 48940 San Sebastian, Spain; juanmaria.blanco@ehu.es; 4 IKERBASQUE, Basque Foundation for Science, 48013 Bilbao, Spain

**Keywords:** amorphous microwires, giant magnetoimpedance effect, magnetoelastic anisotropy, thermal treatment, internal stresses, induced magnetic anisotropy

## Abstract

Magnetic microwires can present excellent soft magnetic properties and a giant magnetoimpedance effect. In this paper, we present our last results on the effect of postprocessing allowing optimization of the magnetoimpedance effect in Co-rich microwires suitable for magnetic microsensor applications. Giant magnetoimpedance effect improvement was achieved either by annealing or stress-annealing. Annealed Co-rich presents rectangular hysteresis loops. However, an improvement in magnetoimpedance ratio is observed at fairly high annealing temperatures over a wide frequency range. Application of stress during annealing at moderate values of annealing temperatures and stress allows for a remarkable decrease in coercivity and increase in squareness ratio and further giant magnetoimpedance effect improvement. Stress-annealing, carried out at sufficiently high temperatures and/or stress allowed induction of transverse magnetic anisotropy, as well as magnetoimpedance effect improvement. Enhanced magnetoimpedance ratio values for annealed and stress-annealed samples and frequency dependence of the magnetoimpedance are discussed in terms of the radial distribution of the magnetic anisotropy. Accordingly, we demonstrated that the giant magnetoimpedance effect of Co-rich microwires can be tailored by controlling the magnetic anisotropy of Co-rich microwires, using appropriate thermal treatment.

## 1. Introduction

Magnetic and magnetoelastic sensors and composites utilizing soft magnetic wires with giant magnetoimpedance (GMI) present extremely high sensitivity to external stimuli, such as magnetic field, stress or temperature, making them suitable for many applications [1,2,3,4,5,6,7]. Most of the emerging applications request reduced dimensionality, combined with excellent soft magnetic properties, superior mechanical properties, enhanced corrosion resistance and biocompatibility [7,8,9,10,11,12]. Generally, amorphous materials, and particularly amorphous magnetic wires, present the best combination of soft magnetic properties and superior mechanical characteristics [13,14]. The other functional properties, like reduced dimensionality, improved corrosion resistance and biocompatibility have been achieved in amorphous microwires coated by flexible and insulating glass-coating [8,9,10,11,12,14]. Therefore, studies of amorphous wires gained considerable attention since the 1970s [1,2,3,4,5,8,9,10]. 

In fact, the Taylor–Ulitovsky technique suitable for preparation amorphous glass-coated microwires with typical metallic nucleus diameters, *d*, ranging between 0.1 and 100 μm (although the most common *d* values are between 5 and 40 μm), coated by thin and continuous glass coating with typical thickness from 0.5 up to 10 μm, was known since the 1960s [15,16]. However, the rediscovery of giant magnetoimpedance, GMI [17,18], (primary discovered in permalloy crystalline wires [19]) stimulated extensive research on the development of magnetically soft wires [20,21,22,23,24]. Particularly, a GMI ratio up to 650% has been achieved in Co-rich glass-coated microwires, either by precise control of the chemical composition and the preparation parameters [25] or by the appropriate postprocessing [26,27,28]. The development of such microwires allows us to achieve extremely high magnetic field sensitivity (up to 10 %/A/m) [26,27,28]. 

The origin of the GMI effect is commonly related to a variation of the skin depth, *δ*, under applied magnetic field, *H*, that can be observed in a magnetic conductor with high circumferential magnetic permeability, *μ_ϕ_*, given by the following equation [17,18,19,20,21,22,23,24]:

(1) $$\delta \;=\;\frac{1}{\sqrt{{\pi \sigma \;\mu }_{\Phi }\;f}}$$

where *σ* is the electrical conductivity and *f* is the AC current frequency.

Accordingly, the magnetic field dependence of the GMI effect is determined by the magnetic field dependence of magnetic permeability, and hence by the type of magnetic anisotropy [17,18,19,20,21,22,23,24,25]. Commonly, the GMI effect is represented by the GMI ratio, Δ*Z/Z*, which is defined as follows:

(2) $$\frac{\Delta Z}{Z}=\frac{Z\;\left(H\right)-{Z(H}_{\max})}{{Z(H}_{\max})}\times 100$$

where *Z* is the sample impedance; and *H* and *H_max_* are the given and maximum applied DC magnetic fields, respectively.

Generally, amorphous wires present soft magnetic properties. However, the best magnetic softness is reported in Co-rich amorphous microwires with nearly-zero magnetostriction coefficient, *λ_s_* [22,23,24,25,26,27,28,29]. The magnetic softness of crystalline magnetic materials is affected by their crystalline structure, various types of defects, grain size and boundaries, texture, etc. In contrast, the soft magnetic properties of amorphous materials are associated to their glassy-like structure and, hence, the absence of the defects typical for crystalline materials [22,24]. Accordingly, the main factors limiting magnetic softness of amorphous materials are the magnetoelastic and shape anisotropies [22,24]. The magnetoelastic anisotropy, *K_me_*, is determined by the magnetostriction coefficient, λ_s_, and the internal stress, σ_i_, [22,24,25]. The magnetostriction coefficient, *λ_s_*, in amorphous alloys is affected by the chemical composition: nearly-zero *λ_s_* values have been reported in Co_x_Fe_1−x_ (0 ≤ x ≤ 1) or Co_x_Mn_1−x_ (0 ≤ x ≤ 1) alloys with Co content, x, ranging between 0.03 and 0.08 [30,31,32]. Therefore, the most common route for optimization of magnetic softness is to use alloys with a vanishing magnetostriction coefficient.

High circumferential magnetic permeability of Co-rich microwires is commonly attributed to the bamboo-like domain structure of the outer domain shell [28,29]. 

Alternatively, vanishing *λ_s_* value and, hence, improved magnetic softness and GMI effect can be achieved by devitrification of quite particular Fe-rich (Finemet-type) wires [32,33,34]. However, the main obstacle for applications of Finemet-type microwires is poor mechanical properties. 

The other parameter that affects the magnetic softness and, hence, the GMI effect of the magnetic microwires is the value and distribution of the internal stresses. In fact, both the value of internal stresses and magnetostriction coefficient are factors that contribute to the magnetoelastic anisotropy [16,25,26,35,36,37,38]. The origins of internal stresses are the rapid melt quenching itself and the different thermal expansion coefficients of the metallic nucleus and the glass coating [35,36,37,38]. Accordingly, for a given chemical composition (with a given magnetostriction), the magnetoelastic anisotropy can be further reduced by internal stresses relaxation.

The most common way of the stress relaxation is the thermal treatment. However, magnetic hardening of Co-rich microwires upon annealing is reported in a few recent publications dealing with attempts to improve their magnetic softness [24,39,40]. The origin of such magnetic hardening was attributed either to the influence of internal stresses on magnetostriction coefficient (and therefore magnetostriction change upon annealing) [39,40,41] or the modification of the domain structure after thermal treatment [41]. However, the beneficial influence of stress-annealing on magnetic softness and the GMI effect is recently reported for Fe-rich microwires with large and positive magnetostriction coefficient [42,43]. A remarkable improvement in magnetic softness and the GMI effect is attributed to stress-annealing-induced transverse magnetic anisotropy [42,43]. 

There are quite a few publications about stress-annealing on magnetic properties and GMI effect of Co-rich microwires [44,45,46]. Among other results, it was observed that, although stress-annealed Co-rich microwires generally showed higher coercivity, *H_c_*, than as-prepared Co-rich microwires, they may have a higher GMI ratio. In addition, stress-annealed Co-rich microwires have a lower *H_c_* and a higher Δ*Z/Z* than Co-rich microwires annealed at the same temperature [44,45,46,47]. Therefore, there are expectations that magnetic hardening previously reported in Co-rich microwires upon annealing can be avoided if conventional annealing will be replaced by stress-annealing.

In this paper, we report experimental results on optimization the GMI effect and magnetic softness of Co-rich glass-coated microwires by annealing and stress-annealing.

## 2. Experimental Methods

As described in the Introduction, magnetic properties and GMI effect of Co-rich microwires depend on the chemical composition of the metallic nucleus and microwires geometry (metallic nucleus, *d*, total diameter, *D*, and their ratio, *ρ = d/D*). Therefore, in order to elucidate the influence of the thermal treatment on hysteresis loops and the GMI effect, we selected just one Co-rich chemical composition and geometry and subjected this microwire to different thermal treatments. 

We prepared amorphous Fe_3.6_Co_69.2_Ni_1_B_12.5_Si_11_Mo_1.5_C_1.2_ (metallic nucleus diameter *d* = 22.8 μm, total diameter *D* = 23.2 μm) glass-coated microwire by using Taylor–Ulitovsky technique, as earlier described [15,16,47,48]. The amorphous character of the samples was checked via X-ray Diffraction (XRD) and by the Differential Scanning Calorimeter (DSC). XRD studies were performed by using a BRUKER (D8 Advance) X-ray diffractometer with Cu K_α_ (λ = 1.54 Å) radiation. XRD spectra of all as-prepared and annealed samples present a broad halo, which is typical for completely amorphous materials. 

For DSC studies, we employed a 204 F1 Netzsch calorimeter. Using the DSC (heating rate of 10 K/min), we determined that the crystallization temperature, *T_cr1_*, (defined as the beginning of the first crystallization peak) in as-prepared microwire is about 553 °C.

The microwires were annealed at a temperature, *T_ann_*, in the range from 200 to 375 °C, in a conventional furnace. Consequently, all the studied microwires keep an amorphous structure and thence presented good mechanical properties typical for amorphous materials. We used a fixed annealing time of 60 min. This annealing time is commonly used for thermal treatment of amorphous and nanocrystalline materials [32,34]. For each annealing temperature, one as-prepared microwire of around 15 cm in length was used. All as-prepared samples selected for studies had the same magnetic properties and geometry (metallic nucleus diameter and glass-coating thickness). All heat treatments were carried out in several samples, and the results were compared, to ensure reproducibility.

Tensile stress was applied during annealing, as well as during cooling of the sample in the furnace. The stress value in the metallic nucleus, σ_m_, was estimated by taking into account the different Young’s moduli of metal, *E*_2_, and glass, *E*_1_, as previously described [43,48]:

(3) $$\sigma_{m}=\frac{K\cdot P}{K\cdot S_{m}+S_{gl}}$$

where *K = E_2_/E_1_*, *P* is the applied mechanical load and *S_m_* and *S_gl_* are the cross-sections of the metallic nucleus and the glass coating, respectively. The value of applied stresses was between 118 and 472 MPa.

**Figure 1:**
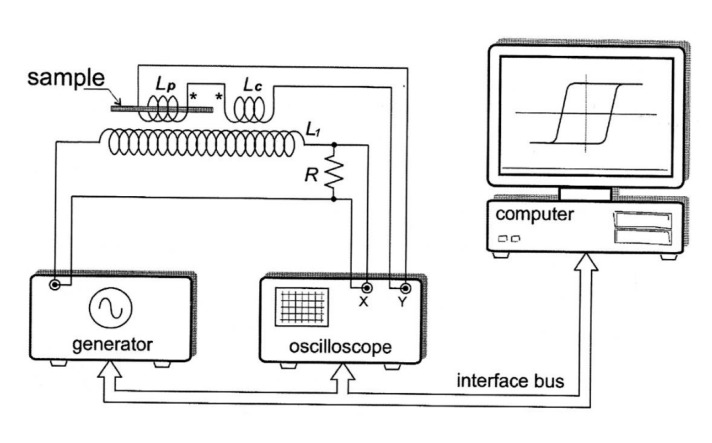
Scheme of the setup allowing measuring of hysteresis loops of magnetic microwires.

Hysteresis loops were recorded by using the fluxmetric method previously successfully employed for studies of magnetic microwires by us [24,25,27]. The schematic picture of the experimental setup is provided in Figure 1. The electromotive force, *ϵ*, in the pick-up coil with *N* turns produced by the change of magnetic flux, *ϕ*, is given by the following equation [29,49]:

(4) $$\epsilon =-N\frac{d\phi }{dt}$$

The microwire occupies a small part of the coil cross-section. Therefore, the magnetic flux produced by the external field can be essentially relevant, and hence it is necessary to consider both parts of magnetic flux, originating from the sample magnetization, *M*, and from the magnetic field, *H*:

(5) $$\phi =\;\mu_{0}\;\left[\left(A_{c}-A_{s}\right)H+\;A_{s}\left(H+M\right)\right]=\;\mu_{0}\left[A_{c\;}H+\;A_{s\;}M\right]$$

where *A_c_* and *A_s_* are the coil and sample cross-section areas. Then, the induced voltage contains two components in pick-up coil.

(6) $$\epsilon =-\;\mu_{0}N\frac{d\left(A_{c}H+\;A_{s}\;M\right)}{dt}=\;-\mu_{0\;}N\;\left[A_{c}\frac{dH}{dt}+A_{s}\frac{dM}{dt}\right]$$

An identical compensation coil is used to eliminate the component *A_c_ (dH/dt)* due to an external magnetic field. The compensation coil is connected in series-opposition with the pick-up coil. Both coils are placed inside the long solenoid coaxially. The distance between the compensation and the pick-up coils is about 7 cm. The compensation quality and lack of interference between the coils are proved by absence of the signal without the sample. The quality of compensation and the absence of interference between the coils are confirmed by the absence of a signal without a sample. Accordingly, the resulting electromotive force, *ϵ_c_*, depends only on the rate of change of the magnetization of the sample, as follows:

(7) $$\epsilon_{c}=-\;\mu_{0}\;N\;A_{s}\frac{dM}{dt}\;$$

As a result, *ϵ_c_* = 0 in the absence of a sample. Then the sample magnetization can be obtained by integrating the induced voltage, as follows:

(8) $$M=\frac{1}{N\mu_{0}A_{s}}\;\int^{\text{​}}\epsilon \;dt$$

The hysteresis loops measurements can be performed at different frequencies, *f*, however, usually *f* = 100 Hz is most useful. As previously shown, this method allows measurements of the hysteresis loops in the *f-*range between 10 and 1000 Hz [50,51]. Generally, the *ϵ_c_* signal grows with frequency increasing. However, at sufficiently high frequencies (*f* > 200 Hz), a change in the overall shape of hysteresis loops is observed: there is a deviation from the perfectly rectangular hysteresis loop typical of a magnetically bi-stable microwire. This change in the hysteresis loop shape was explained by considering the counterbalance between the sweeping rate, *dH/dt*, and the switching time related to the time of domain wall propagation throughout the wire. Therefore, the frequency of about 100 H_Z_ was selected.

Hysteresis loops can be represented as the normalized magnetization, *M/M_o_*, versus the applied magnetic field, *H*, where *M_o_* is the magnetic moment of the sample at the maximum magnetic field amplitude, *H_o_* [49]. 

The microwire impedance, *Z*, was evaluated from the reflection coefficient, *S_11_,* and measured using a vector network analyzer and a micro-strip sample holder, as described elsewhere [52,53]. A previously developed method allowed *Z* measurements within the wide frequencies, *f*, up to GHz frequencies [52]. The use of a sufficiently long solenoid with micro-strip sample holder placed inside allowed us to measure the magnetic field dependence of the GMI effect. The GMI ratio was evaluated by using the Equation (2).

Furthermore, the magnetostriction coefficient, λ_s_, of the studied microwire, estimated using the SAMR method adapted for microwire research, as described elsewhere [31,54], gives a value of about λ_s_ ≈ −0.3 × 10^−6^.

## 3. Results and Discussion

**Figure 2:**
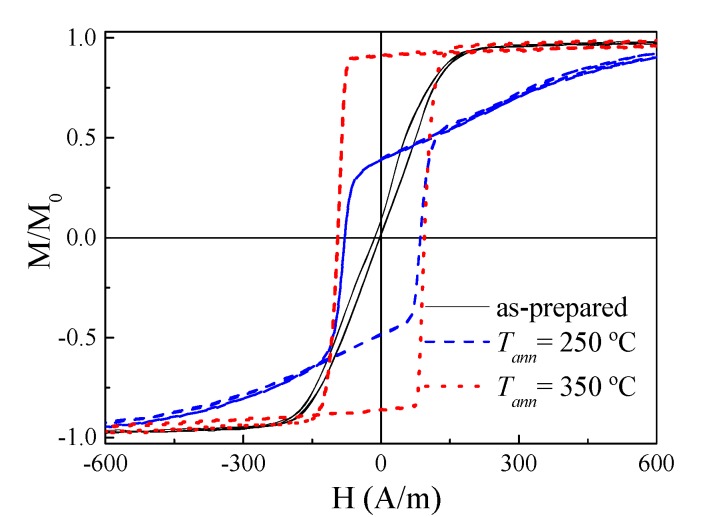
Hysteresis loop of as-prepared and annealed without stress at different *T_ann_* for *T_ann_* = 60 min samples.

As-prepared Fe_3.6_Co_69.2_Ni_1_B_12.5_Si_11_Mo_1.5_C_1.2_ microwire exhibits linear hysteresis loop with low coercivity (*H_c_* ≈ 4 A/m, see Figure 2). Similar to what is reported for the other Co-rich microwires with similar *λ_s_* values [39,40], perfectly rectangular hysteresis loops with coercivity, *H_c_* ≈ 90 A/m, were observed in samples annealed at sufficiently high *T_ann_* (see Figure 2). 

**Figure 3:**
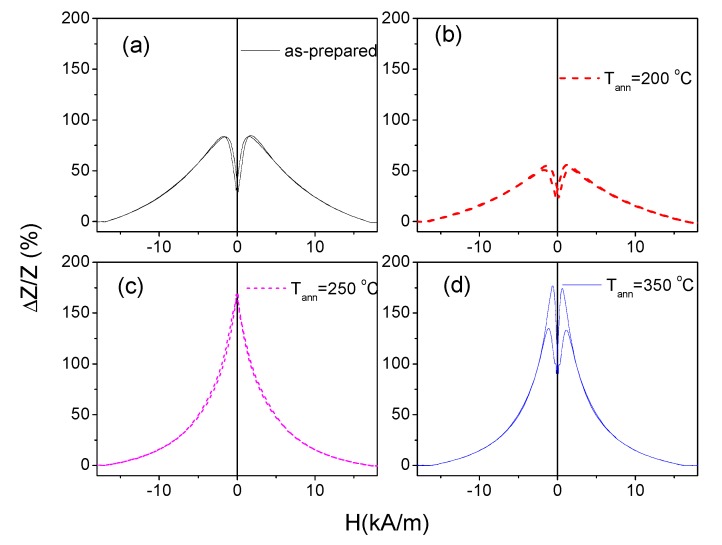
ΔZ/Z(H) dependencies of as-prepared (**a**) and annealed at *T_ann_* = 200 °C (**b**), *T_ann_* = 250 °C (**c**) and *T_ann_* = 350 °C (**d**) samples measured at 200 MHz.

All annealed samples present almost the same coercivity. However, increase of the squareness ratio, *M_r_/M_max_*, upon *T_ann_* rising can be appreciated (Figure 2). As recently reported [46], Co-rich microwires present maximum GMI ratio at frequencies, *f*, about 100–200 MHz. Therefore, the comparative studies of annealing temperature effect on the GMI ratio are performed for *f* = 200 MHz. As can be appreciated from Figure 3, the maximum GMI ratio, Δ*Z/Z_m_*, decreases after annealing at *T_ann_* = 200 °C. However, samples annealed at higher *T_ann_* exhibit larger Δ*Z/Z_m_* ratios (see Figure 3c,d). 

Moreover, annealing affects not only the Δ*Z/Z_m_* values, but also the shape of Δ*Z/Z(H)* dependencies: as-prepared exhibits double-peak Δ*Z/Z(H)* dependence (see Figure 3a). However, a noticeable modification of the Δ*Z/Z (H)* dependencies can be appreciated upon annealing: For the samples annealed at *T_ann_* = 200 and 350 °C, Δ*Z/Z(H)* dependencies still present a double-peak shape. However, the magnetic field at which the maximum on Δ*Z/Z(H)* dependence takes place, *H_m_*, becomes lower than that for the as-prepared sample (*H_m_* ≈ 1.7 kA/m for as-prepared sample, *H_m_* ≈ 1.2 kA/m for T_ann_ = 200 °C and *H_m_* ≈ 0.7 kA/m for *T_ann_* = 350 °C; see Figure 3a,b,d). 

Finally, the Δ*Z/Z(H)* dependence in the samples annealed at *T_ann_* = 250 °C present a decay with magnetic field increase from *H* = 0 (Figure 3c). 

The observed annealing influence on Δ*Z/Z(H)* dependencies correlates with the evolution of the hysteresis loops upon annealing and hence can be associated with internal stresses relaxation [28,29,30,53]. Within the framework of the core–shell model of the domain structure of amorphous ferromagnetic wires, the inner axially magnetized core radius, *R_c_*, can be estimated from the squareness ratio, *M_r_/M_o_*, as follows [28,29,30,55]:

(9) $$R_{C}\;=\;R\sqrt{\frac{M_{r}}{M_{0}}}$$

where *R* is the microwire radius. 

As can be observed in Figure 2, squareness ratio, *M_r_/M*_0_, rapidly increases upon annealing.

**Figure 4:**
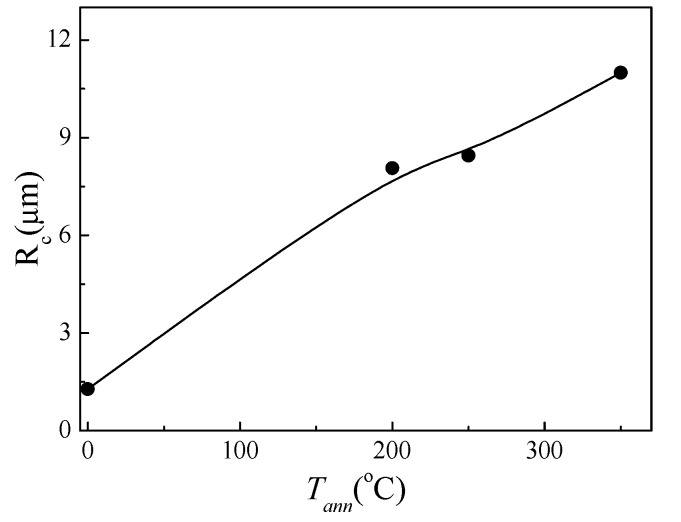
*R_c_*(*T_ann_*) dependence evaluated from hysteresis loops of studied microwire.

Accordingly, from *R_c_*(*T_ann_*) dependence evaluated by using Equation (9), we can deduce that the inner axially magnetized core radius, *R_c_*, increases after annealing, as shown in Figure 4. 

Furthermore, at *T_ann_* = 350 °C, *R_c_* ≈ 0.97R, i.e., almost entire sample volume consists of the axially magnetized core. Accordingly, axial magnetic anisotropy can be considered for annealed samples. Previously, the arising of rectangular hysteresis loops and, hence, axial magnetic anisotropy was explained by internal stresses relaxation, as well as the magnetostriction coefficient modification upon annealing [39,40,41].

The magnetic field dependence of impedance, Z, is determined by the type of magnetic anisotropy [21]. The decrease in *Z(H)* from *H* = 0 is reported for magnetic wires with axial magnetic anisotropy [21,22]. The double-peak *Z(H)* dependencies are predicted and observed for magnetic wires with transverse magnetic anisotropy [21,22,23,41]. Therefore, the observed modification of Δ*Z/Z(H)* dependencies upon annealing (see Figure 3) correlates well with the evolution of the bulk hysteresis loops.

**Figure 5:**
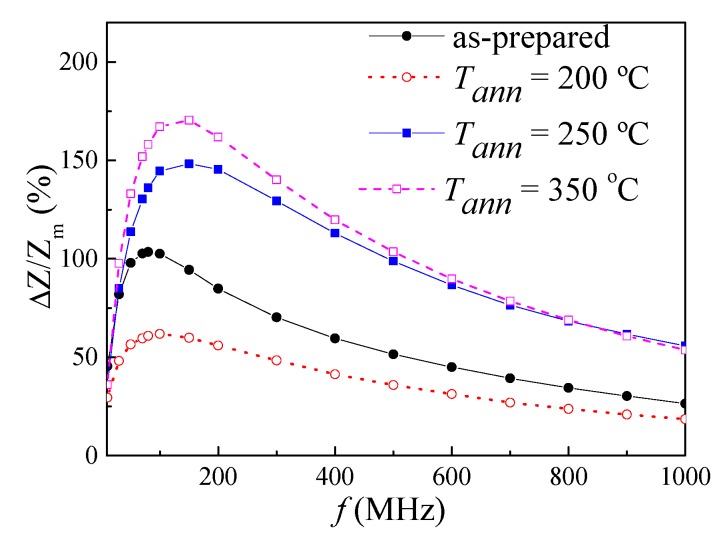
Frequency dependence of the maximum GMI ratio evaluated for as-prepared and annealed at different *T_ann_* microwires.

As can be observed from Figure 5, the evolution of Δ*Z/Z_m_* upon annealing is not restricted to 200 MHz: Surprisingly, higher Δ*Z/Z_m_* values for *T_ann_* = 250 and 350 °C are observed in a wide frequency range. Previously, decreasing of the GMI ratio in annealed Co-rich microwires presenting with annealing-induced magnetic bistability has been reported [39]. However, systematic studies have not been conducted.

The observed GMI ratio improvement upon annealing can be explained by the high circumferential magnetic permeability in the surface layer responsible for the GMI effect. As can be deduced from Figure 4, the samples annealed at *T_ann_* = 200 and 250 °C present rather similar *R_c_* values (8.1 and 8.5 µm, respectively). However, lower Δ*Z/Z_m_* values are observed for the sample annealed at *T_ann_* = 200 °C. Therefore, the reason for lower Δ*Z/Z_m_* of the samples annealed at *T_ann_* = 200 °C can be related to deeper internal stresses relaxation and, hence, higher circumferential magnetic permeability in the surface layer upon annealing at *T_ann_* = 250 °C.

As mentioned in the introduction, transverse magnetic anisotropy can be induced by stress-annealing, at least in Fe-rich microwires [42,43]. Such transverse magnetic anisotropy allowed remarkable magnetic softening and the GMI-effect improvement. Additionally, higher GMI effect in some frequency ranges has been reported for Co-rich microwires, too [44]. Therefore, we studied the influence of stress-annealing on the GMI effect. 

**Figure 6:**
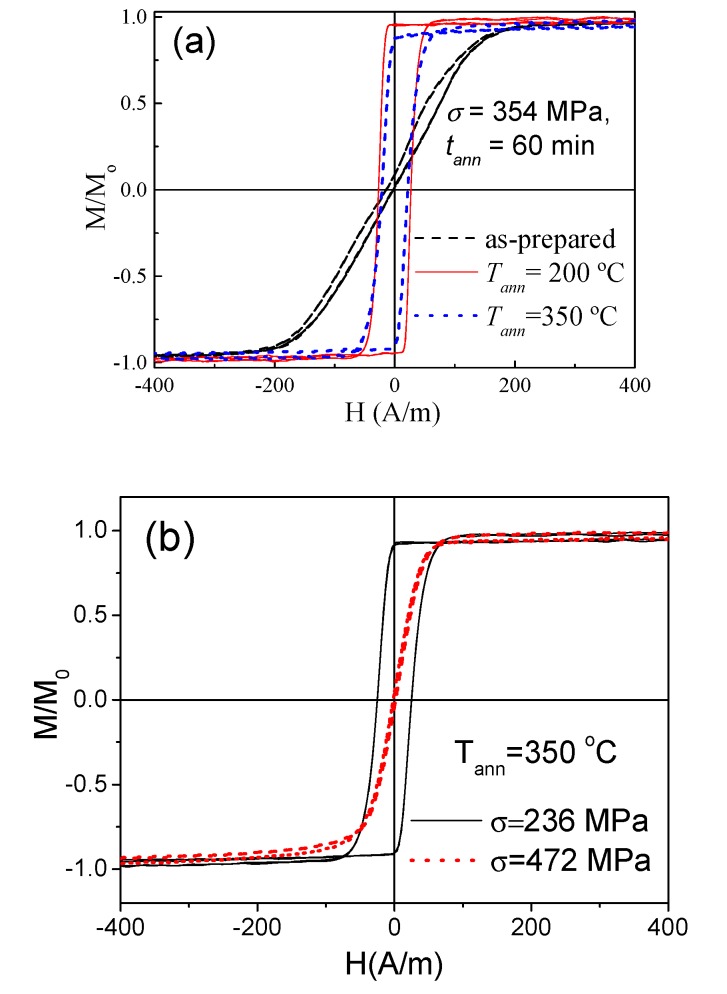
Effect of *T_ann_,* for *σ* = 354 MPa (**a**) and effect of *σ* for *T_ann_* = 350 °C (**b**), on hysteresis loops of studied samples.

Influence of various parameters, like annealing temperature and stress applied during the annealing on hysteresis loops of the studied samples is provided in Figure 6.

From the hysteresis loops provided in Figure 2 and Figure 6, we can deduce that, similar to the case of Fe-rich microwires, stress-annealing allows for the induction of transverse magnetic anisotropy. However, rather higher *T_ann_* and σ values are needed to induce transverse magnetic anisotropy in studied Co-rich microwires. For intermediate *T_ann_* and *σ* values, stress-annealed microwires present lower coercivity (20 ≤ *H_c_* ≤ 25 A/m for 200 °C ≤ *T_ann_* ≤ 350 °C and *σ* ≤ 354 MPa) and higher squareness ratio (*M_r_/M_o_* ≈ 0.97 for 200 °C ≤ *T_ann_* ≤ 350 °C and *σ* ≤ 354 MPa). For comparison, remarkable transverse-stress-annealing-induced magnetic anisotropy has been reported for Fe_75_B_9_Si_12_C_4_ microwire annealed at *T_ann_* = 300 °C (*σ* = 380 MPa) or *T_ann_* = 325 °C (*σ* = 190 MPa) [49].

For sufficiently high *T_ann_* and *σ* (*σ* = 472 MPa *T_ann_* = 350 °C), we obtained samples with an almost-linear hysteresis loop, extremely low coercivity (*H_c_* ≈ 2 A/m), squareness ratio (*M_r_/M_max_* < 0.1) and magnetic anisotropy field (*H_k_* ≈ 70 A/m) (see Figure 6b). 

**Figure 7:**
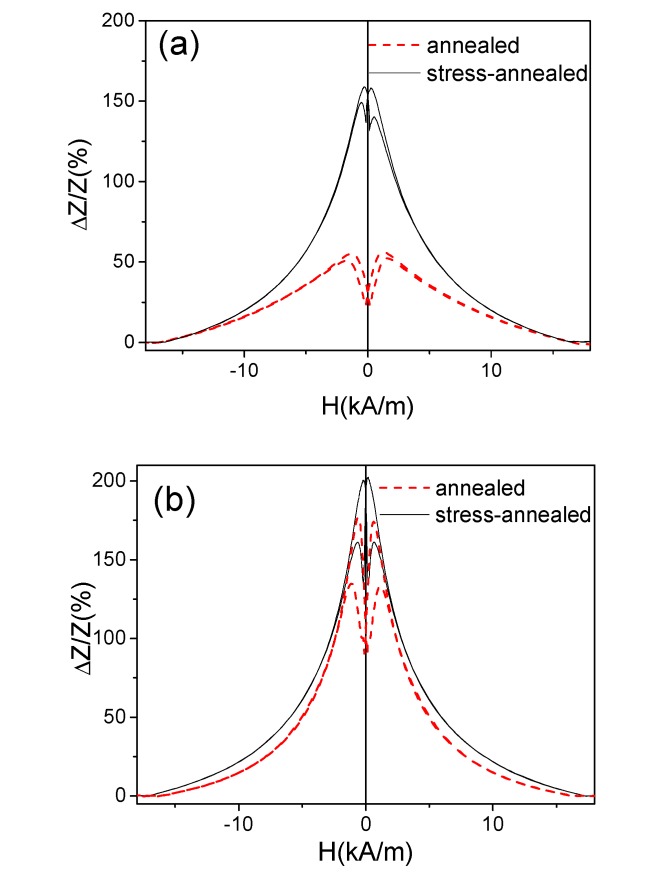
Comparison of Δ*Z/Z(H)* dependencies of annealed and stress-annealed (*σ* = 354 MPa) at *T_ann_* = 200 °C (**a**) and *T_ann_* = 350 °C samples (**b**) measured at 200 MHz.

A comparison of the Δ*Z/Z(H)* dependencies of annealed and stress-annealed at the same annealing temperatures (*T_ann_* = 200 °C and *T_ann_* = 350 °C) for microwires measured at the same frequency (200 MHz) is provided in Figure 7. In both cases, stress-annealed samples present considerably higher Δ*Z/Z_m_* values. However, this difference is more remarkable for *T_ann_* = 200 °C (see Figure 7a).

**Figure 8:**
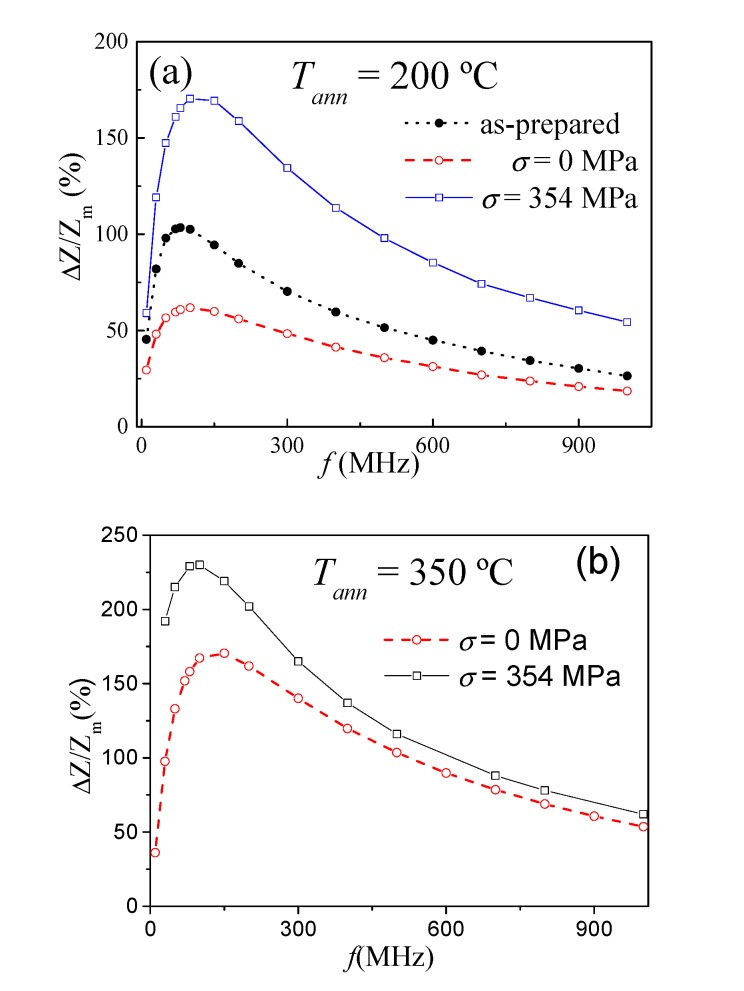

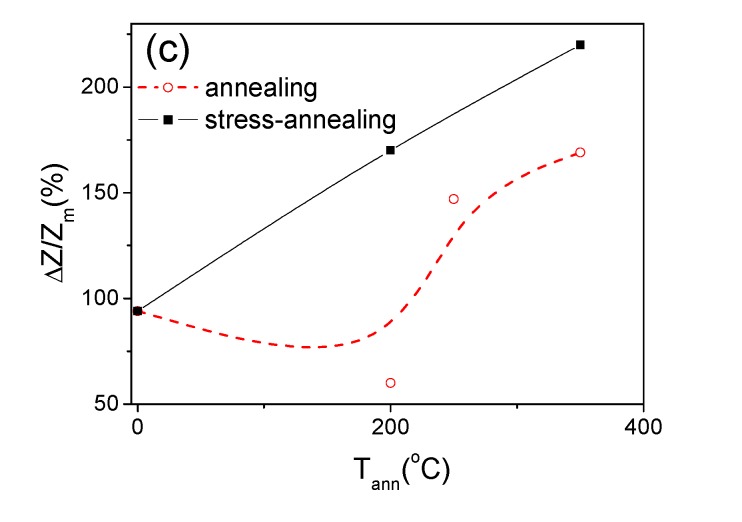
Δ*Z/Z_m_ (f)* dependencies evaluated for annealed and stress-annealed samples at *T_ann_* = 200 °C (**a**) and *T_ann_* = 350 °C (**b**) and Δ*Z/Z_m_(T_ann_)* dependence (**c**) evaluated for *f* = 150 MHz for annealed and stress-annealed samples. The lines are just to guide the eyes.

As can be appreciated from Figure 8, Δ*Z/Z_m_* improvement is observed for the whole frequency range for both *T_ann_*. Similar to the as-prepared sample, the highest Δ*Z/Z_m_* values are observed between 100 and 200 MHz.

The observed influence of postprocessing on the GMI effect of studied Co-rich microwires can be summarized as follows: (i) appropriate postprocessing (annealing or stress-annealing) can be beneficial for GMI effect improvement; (ii) application of stress during annealing allows remarkable decrease of coercivity and increase of squareness ratio at moderate σ and *T_ann_* values; (iii) transverse magnetic anisotropy can be induced by stress-annealing at sufficiently high *σ* and *T_ann_* values; (iv) creep-induced magnetic anisotropy depends on *σ* for *T_ann_* values; and (v) observed GMI ratio improvement is observed in the whole frequency range employed in these studies.

A more remarkable influence of stress-annealing can be appreciated from Figure 8c, where Δ*Z/Z_m_(T_ann_)* dependence evaluated for *f* = 150 MHz for annealed and stress-annealed samples is shown. ∆*Z/Z_m_* varies from 94% for as-prepared up to 220% for stress-annealed.

One of the unusual results is that the GMI ratio improvement is observed even for annealed samples which present perfectly rectangular hysteresis loops. As reported recently, Co-rich microwires annealed and even stress-annealed at moderate σ and *T_ann_* values present single and fast domain wall propagation [46,47]. Consequently, the existence of inner axially magnetized single domain core must be assumed for Co-rich microwires annealed and stress-annealed at moderate σ and *T_ann_* values. Our evaluation of the inner axially magnetized core radius (see Figure 4) gives values up to 95% for the total metallic nucleus volume. However, improved GMI ratio of annealed and stress-annealed samples must be associated to the existence of surface layer with high circumferential magnetic permeability.

Δ*Z/Z(H)* dependencies of annealed and stress-annealed microwires measured at 200 MHz (Figure 3c,d and Figure 7) are consistent with their axial magnetic anisotropy deduced from bulk hysteresis loops. On the other hand, GMI effect is essentially restricted to the magnetic properties in the surface layer of microwires. The skin depth, *δ*, given by Equation (1) is affected by a few parameters, among them, the frequency, *f*: By raising the frequency, *f*, the minimum skin depth, *δ_m_*, decreases [56]. 

Consequently, the frequency dependence of the GMI ratio must be related to the radial distribution of the magnetic anisotropy: At higher frequencies, thinner surface layers must be involved in the Δ*Z/Z(H)* dependencies.

**Figure 9:**
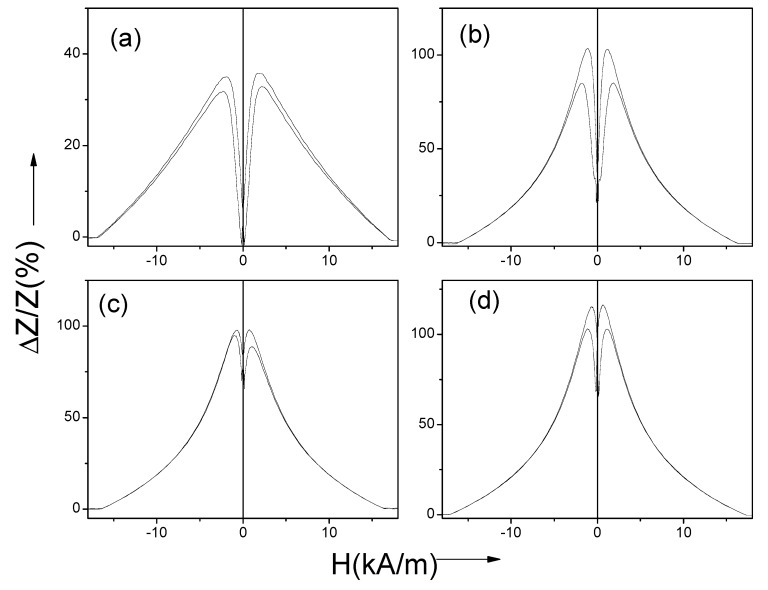
Δ*Z/Z(H)* dependencies measured at 500 MHz, annealed at *T_ann_* = 200 °C and *T_ann_* = 350 °C (**a**,**b**) and stress-annealed at the same *T_ann_* (**c**,**d**) microwires.

As observed in Figure 9, all annealed and stress-annealed samples measured at 500 MHz present double-maximum Δ*Z/Z(H)* dependencies, which are typical for transverse magnetic anisotropy. This difference in Δ*Z/Z(H)* dependencies for 200 and 500 MHz can be related to the circumferential magnetic anisotropy of the thin surface layer of the studied samples. 

In fact, the penetration skin depth, *δ*, and its dependence on magnetic field and frequency can be evaluated from the Δ*Z/Z(H)* dependencies, considering that the changes in the real component of the impedance are due to changes in the effective area in which the AC-current flows as a consequence of the skin-effect [56,57,58,59,60]. Such an approach relates the penetration depth, *δ*, and the ratio *R_DC_/R_AC_* (*R_DC_* is the DC-resistance of the wire, and *R_AC_* is the real component of the impedance) as follows:

*δ* = *r*[1 − (1 − *R_DC_*/*R_AC_*)^1/2^] (10)

where r is the wire radius.

In the case of stress-annealed FeSiBC microwires, a drastic decrease of minimum penetration depth, *δ*_min_, upon stress-annealing has been reported [57].

**Figure 10:**
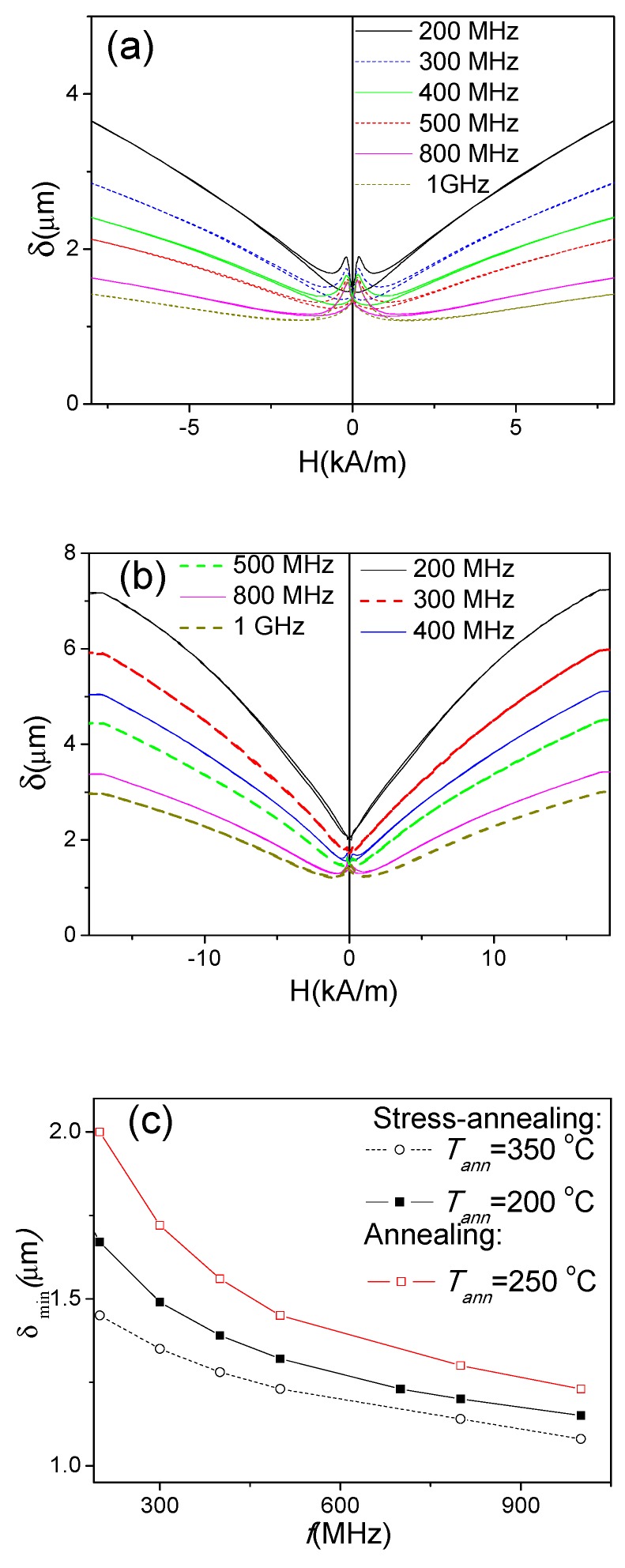
Calculated *δ(H)* dependencies for stress-annealed at *T_ann_* = 350 °C sample (**a**) for the sample annealed (without stress) at *T_ann_* = 250 °C (**b**) and *δ_min_(f)* dependencies estimated for stress-annealed at *T_ann_* = 350 °C and *T_ann_* = 200 °C samples (**c**).

The evaluation of *δ (H)* dependencies for the sample stress-annealed at 350 °C, presented in Figure 10a, shows features similar to those reported for as-prepared Co-rich microwires and as-prepared and stress-annealed Fe-rich microwires: a noticeable dependence on the magnetic field and the frequency. Similarly, the *δ (H)* dependencies for the sample annealed at 250 °C (without stress) are presented in Figure 10b. The *δ (H)* dependencies of the annealed and the stress-annealed samples present similar features. However, the stress-annealed samples have lower *δ*_min_ values.

The frequency dependence of the minimum penetration depth, *δ*_min_, evaluated from Figure 10a,b, shows a decrease with *f* rising. The sample stress-annealed at *T_an_* = 200 °C presents similar *δ*_min_ *(f)* dependence, with slightly higher *δ*_min_ values (see Figure 10c): In both stress-annealed samples, *δ*_min_ values near 1–1.2 μm can be observed at high frequencies. Finally, the sample annealed without stress presents slightly higher *δ*_min_ values, of about 1.3 μm.

On the other hand, the approximate thickness of the outer domain shell with transverse magnetic anisotropy, estimated from Figure 4 (where the estimation of the inner axially magnetized domain radius is provided), gives values of about 0.5 μm. It is clear that, if *δ*_min_ becomes comparable with the outer domain shell thickness, its influence can be more significant.

Accordingly, improvement of the GMI ratio observed in annealed and stress-annealed samples exhibiting rectangular hysteresis loops and observed modification of the Δ*Z/Z(H)* dependencies with frequency can be attributed to the spatial distribution of the magnetic anisotropy. In particular, the existence of an inner axially magnetized core and an outer shell with high circumferential magnetic permeability near the surface can be assumed for annealed and stress-annealed microwires. Discussed radial distribution of the magnetic anisotropy can be attributed to the stress-induced anisotropy. 

The origin of stress-induced anisotropy in amorphous materials is still not clear, although it is discussed in numerous publications [60,61,62]. The most common origin of stress-annealing-induced anisotropy is either “back stresses” or directional pair (chemical or topological) ordering [41,42,43,44,45,46,47,48,49,60,61,62]. 

The pair-ordering mechanism is commonly considered for amorphous alloys with two or more magnetic elements. Accordingly, for studied multicomponent Fe_3.6_Co_69.2_Ni_1_B_12.5_Si_11_Mo_1.5_C_1.2_ microwire, this mechanism can be considered. However, as mentioned above, in the case of studied microwires, higher *T_ann_* and *σ* (as compared to Fe-rich microwires) are requested to observe considerable transverse magnetic anisotropy [41,42,43,44,45,46,47,48,49]. Therefore, observed stress-annealing-induced anisotropy can present similar origins for the case of Fe-rich microwires, i.e., either back stresses or topological short-range ordering [41,42,43,44,45,46,47,48,49]. The particularity of glass-coated microwires is that they are essentially composites consisting of metallic alloy nucleus and glass-coating that induces strong internal stresses [35,36,37,38,39]. Consequently, as previously proposed elsewhere [63,64], “back stresses” can appear during the annealing and subsequent cooling.

On the other hand, the alternative origin of the radial distribution of magnetic anisotropy and different magnetic anisotropy in the surface layer of metallic nucleus was recently attributed to the existence of the interfacial layer between the metallic nucleus and the glass coating [65,66]. 

As can be seen from the observed dependences, both annealing and stress-annealing are promising methods for optimization the GMI ratio of Co-rich magnetic microwires.

The above examples provide routes to optimize the GMI effect in Co-rich microwires.

## 4. Conclusions

We have demonstrated that the GMI effect of Co-rich microwires can be remarkably improved by appropriate thermal treatment. 

A significant improvement in the GMI ratio at certain annealing conditions is observed in spite of remarkable magnetic hardening and transformation of a linear hysteresis loop with low coercivity (*H_c_* ≈ 4 A/m) to a rectangular one with *H_c_* ≈ 90 A/m upon annealing of Co-rich microwires. The GMI effect can be further improved by the stress-annealing.

The hysteresis loops of stress-annealed microwires are considerably affected by the stress-annealing conditions (annealing time, temperature or stress applied during the annealing).

Stress-annealing performed at moderate values of annealing temperatures and stress allows for a remarkable decrease of coercivity and increase of squareness ratio and further GMI-effect improvement. Stress-annealing, carried out at sufficiently high temperatures and/or stress allowed induction of transverse magnetic anisotropy, as well as GMI-effect improvement. Frequency and magnetic field dependencies of penetration skin depth were evaluated from Δ*Z/Z(H)* dependencies. 

Enhanced GMI ratio values for annealed and stress-annealed samples, and the evolution of Δ*Z/Z(H)* dependencies with frequency and dependence of penetration skin depth on frequency and magnetic field were discussed in terms of the radial distribution of the magnetic anisotropy.

Consequently, the GMI effect of Co-rich microwires can be optimized by the appropriate postprocessing.
